# Mesoionic compounds with antifungal activity against *Fusarium verticillioides*

**DOI:** 10.1186/s12866-015-0340-9

**Published:** 2015-02-04

**Authors:** Rojane de Oliveira Paiva, Lucimar Ferreira Kneipp, Camilla Moretto dos Reis, Aurea Echevarria

**Affiliations:** Departamento de Química, Instituto de Ciências Exatas, Universidade Federal Rural do Rio de Janeiro, 23890-000 Seropédica, RJ Brazil; Laboratório de Taxonomia Bioquímica e Bioprospecção de Fungos, Instituto Oswaldo Cruz, Fiocruz, Rio de Janeiro, 21040-900 RJ Brazil

**Keywords:** Mycotoxigenic fungi, Mycotoxicology, Mesoionic, Antifungal activity, Ergosterol

## Abstract

**Background:**

Fungi contaminate the food of humans and animals, are a risk to health, and can cause financial losses. In this work, the antifungal activities of 16 mesoionic compounds (**MI 1–16**) were evaluated against mycotoxigenic fungi, including *Aspergillus* spp.*, Fusarium verticillioides* and *Penicillium citrinum*. Furthermore, the decreased ergosterol in the total lipid content of *Fusarium verticillioides* was investigated.

**Results:**

*F. verticillioides* was the most sensitive fungus to the mesoionic compounds. Among the evaluated compounds, **MI-11** and **MI-16** presented higher antifungal effects against *F. verticillioides*, with MIC values of 7.8 μg/ml, and **MI-2** and **MI-3** followed, with MICs of 15.6 μg/ml. The most active compounds were those with heterocyclic ring phenyl groups substituted by electron donor moieties (**MI-11** and **MI-16**). Among some compounds with higher activity (**MI-2**, **MI-11** and **MI-16**), decreased ergosterol content in the total lipid fraction of *F. verticillioides* was demonstrated. **MI-2** reduced the ergosterol content approximately 40% and 80% at concentrations of 7.8 μg/ml and 15.6 μg/ml, respectively, and **MI-11** and **MI-16** decreased the content by 30% and 50%, respectively, when at a concentration of 7.8 μg/ml.

**Conclusion:**

These findings indicate that mesoionic compounds have significant antifungal activity against *F. verticillioides.*

## Background

Filamentous fungi produce mycotoxins, which are secondary metabolites that cause mycotoxicosis when ingested by higher animals. Mycotoxins may contaminate cereal plants through the fungi growing on those plants in two ways: as pathogens on plants or saprophytically on stored plants [1]. Fungal growth in cereals and grain in fields as well as stored grain causes nutritional and physical losses. Fungal growth could negatively impact animal performance due to the production of mycotoxins, which are highly toxic to animal and humans [2]. Furthermore, agricultural commodities could be contaminated in the field or during storage by various fungal species [3].

The growth and production of mycotoxins by fungi of the genera *Aspergillus*, *Penicillium* and *Fusarium* are unavoidable under certain environmental conditions, although the prevention of mycotoxin contamination of various commodities in the field is the main goal of the agricultural and food industries [4]. The mycotoxins produced by *Aspergillus* species include aflatoxin, considered very potent liver carcinogens in various animal species and humans. *Penicillium* species can produce ochratoxin, patulin, and citrinin [2], and *Fusarium* species produce fumonisins that can cause a variety of health problems in animal species, including humans and leukoencephalomalacia in horses [5]*.* Thus, the search for new and safer compounds with antifungal activity is important and necessary.

Mesoionic compounds are a special class of heterocyclic compounds having various biological activities such as antifungal, antitumor activities among other [6-9]. Possessing a betaine-like character with positive charge in a polyheteroatomic system and negatively charged atom or exocyclic group, these compounds are able to interact with biomolecules. In addition, their overall neutrality allows them to cross biological membranes [10].

In this work, considering the special chemical structure of the 1,3,4-thiadiazolium-2-aminide mesoionic class, the antifungal activities of 16 mesoionic compounds (**MI 1–16**) were evaluated against mycotoxigenic fungi, i.e., *Aspergillus flavus, A. nomius, A. ochraceus, A. parasiticus, Fusarium verticillioides* and *Penicillium citrinum*. Furthermore, *F. verticillioides*, which was the most sensitive fungus to the mesoionic compounds, showed a decreased amount of ergosterol in its total lipid content.

## Results and discussion

Several methods for the prevention and control of hazardous fungi and their dangerous mycotoxins have been presented [11]. The prevention of fungal invasion of commodities is by far the most effective method of avoiding mycotoxin problems. Despite these advances, the design of new compounds to address the resistance to available drugs has become a major effort in an attempt to solve this public health problem. Among the chemical approaches, different types of compounds, such as those containing a thiadiazole group, have been designed and synthesized, and many of them exhibit broad-spectrum biological activities, such as, antibacterial activity against *Escherichia coli, Micrococus luteus* and *Staphylococcus aureus,* antifungal activity against *Curvuliaria lunata*, *A. flavus* and *A. niger* [6], anti-inflammatory and anticancer activities [12]. It was also demonstrated that these compounds present low toxicity *in vitro* [12]. In this context, the significant antifungal activity has encouraged further studies on mesoionic derivatives containing the thiadiazole moiety, still no reference in literature.

**Figure 1:**
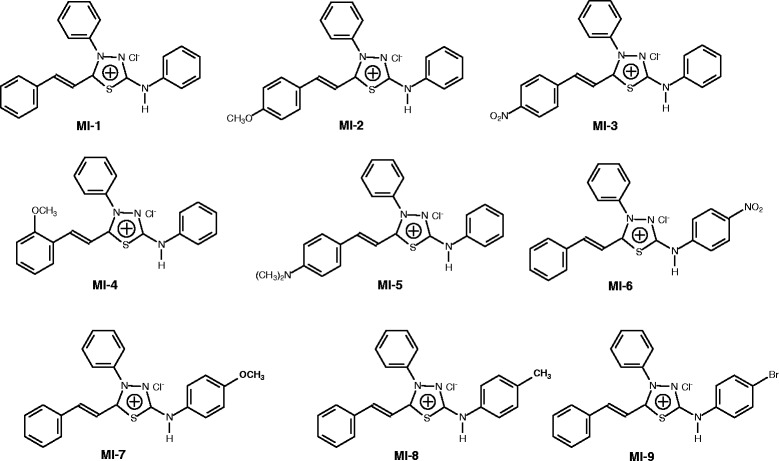
**Chemical structures of the mesoionic derivatives with styryl moieties, MI 1–9 (series I).**

In this work, the antifungal activity of sixteen (16) compounds of the mesoionic class, namely 4-phenyl-5-(4’-X-styryl)-1,3,4-thiadiazolium-2-phenylamine chloride (series I, **M-1** to **M-9**) and 4,5-diphenyl-1,3,4-thiadiazolium-2-(4’-Y-phenylamine) chloride (series II, **M-10** to **M-16**), were evaluated against different mycotoxigenic fungi. The mesoionic derivatives were synthesized in accordance with the literature using a green chemistry method that utilized microwave irradiation and solvent-free conditions and resulted in good yields within a range of 90-98% [9]. The evaluated compounds could be grouped based on structural characteristics into two groups. The first group, series I, consisted of compounds with a styryl group (**MI 1–9**); one was unsubstituted (**MI-1**), four were substituted (**MI 2–5**), and four had substituents on the aromatic ring attached to the exocyclic nitrogen atom (**MI 6–9**), as shown in Figure 1.

**Figure 2:**
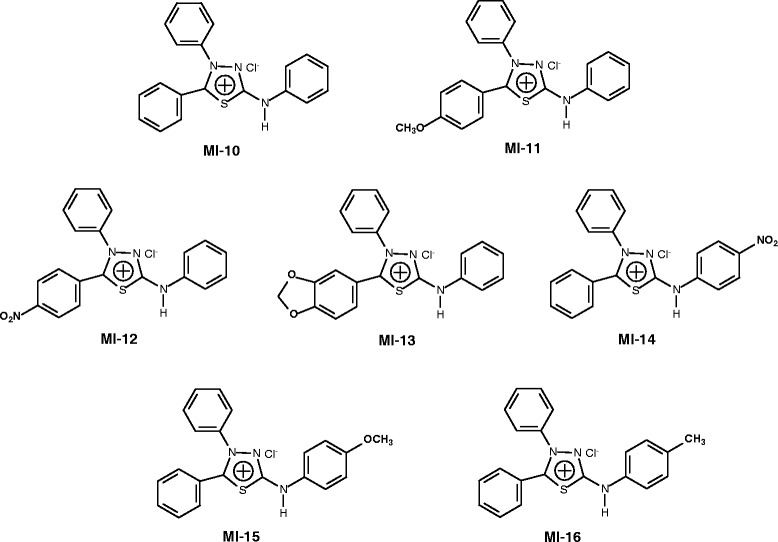
**Chemical structures of the mesoionic derivatives with phenyl substitutions, MI 10–16 (series II).**

The second mesoionic derivative group, series II, had substituents on its phenyl ring attached to the 5-carbon atom (**MI 10–16**); one was unsubstituted (**MI-10**), three had a 5-phenyl substitution (**MI 11–13**), and three had a 2-phenyl substitution and ring attached to the exocyclic nitrogen atom (**MI 14–16**), as shown in Figure 2.

The mesoionic compounds were characterized using ^1^H and ^13^C NMR and IR spectroscopies. The ^1^H and ^13^C chemical shifts were assigned based on literature data [9,8], and were consistent with the structures proposed. The chemical shifts assigned to hydrogens linked to exocyclic nitrogen were observed in the range of 12.30-1.62 ppm, and the vinylic hydrogens in the range of 7.83-8.25 and 7.04-7.25 to H-α and H-β, respectively. The chemical shifts of heterocyclic carbons in ^13^C NMR spectra were assigned to C-5 and C-2 in the range of 156.0-165.8 ppm and 148.5-160.5 ppm, respectively.

The mesoionic compounds (**MI 1–16)** were assayed against *A. flavus, A. nomius, A. ochraceus, A. parasiticus, F. verticillioides* and *P. citrinum* using the broth microdilution test [13]. The results of the antifungal activity evaluation of the mesoionic derivatives are shown in Table 1 and revealed that *F. verticillioides* was the most sensitive to the action of these compounds. Among the assayed compounds, ten were effective against *F. verticillioides,* with MIC values ranging from 7.8 to 125 μg/ml. The best MIC values for the *F. verticillioides* tests were for compounds **MI-11** and **MI-16**, which had MICs of 7.8 μg/ml; followed by **MI-2** and **MI-3**, with MICs of 15.6 μg/ml; **MI-1**, **MI-5**, **MI-14** and **MI-15**, with MICs of 31.2 μg/ml; **MI-12**, with an MIC of 62.5 μg/ml; and **MI-13**, with an MIC of 125 μg/ml*.*

**Table 1:** **MIC/MFC values (μg/ml) of the mesoionic compounds (MI 1–16) against mycotoxigenic fungi**

**Compound A**	***flavus A.***	***nomius A.***	***ochraceus A.***	***parasiticus A.***	***verticillioides F.***	***P. citrinum***
**MI-1**	500	500	500	500	31.2/62.5^a^	500
**MI-2**	500	500	500	500	15.6/125^a^	500
**MI-3**	500	500	>500	500	15.6/125^a^	>500
**MI-4**	>500	>500	>500	>500	>500	>500
**MI-5**	250	250	>500	250	31.2/125^a^	>500
**MI-6**	>500	>500	>500	>500	>500	>500
**MI-7**	>500	>500	>500	>500	>500	>500
**MI-8**	>500	500	>500	500	>500	>500
**MI-9**	>500	>500	>500	>500	>500	>500
**MI-10**	>500	500	>500	500	>500	>500
**MI-11**	500	500	500	500	7.8/62.5^a^	>500
**MI-12**	500	500	500	500	62.5/n.d.^b^	>500
**MI-13**	500	250	500	500	125/n.d.^b^	500
**MI-14**	500	500	250	500	31.2/125^a^	500
**MI-15**	500	500	>500	500	31.2/250^a^	>500
**MI-16**	500	500	500	500	7.8/125^a^	>500
**Itraconazole** ^**c**^	0.12	0.12	0.12	0.12	4	0.12

The MIC was determined as recommended by the CLSI document [24]. >500 represents that concentrations above 500 μg/ml did not affect fungal growth. ^a^MFC values in μg/ml. ^b^n.d.: not determined. ^c^Reference compound.

*F. verticillioides* as well as other species of the *Fusarium* genus produce fumonisins. *F. verticillioides* is one of the main contaminants of corn grains around the world [14-16]. In Brazil, *F. verticillioides* commonly occurs and frequents seeds and grains of maize produced in all regions [17,18]. The literature does not possess records for the evaluation of antifungal activity against *Fusarium* species with mesoionic compounds; however, some studies on thiadiazole-moiety compounds have been reported [12,19]. Comparing the *F. verticillioides* MIC values to the most-active compounds showed that the methoxy- and methyl-substituted derivatives of series II (**MI-11** and **MI-16**) had a higher antifungal effect, with MIC values of 7.8 μg/ml (19.60 μM), which indicated the importance of the donor electron.

The *Aspergillus* genus was less sensitive to the assayed compounds. Among the tested mesoionic derivatives, **MI-5** had an MIC of 250 μg/ml for *A. flavus, A. nomius* and *A. parasiticus*, and **MI-13** and **MI-14** presented the same MIC values for *A. nomius* and *A. ochraceus.***MI-6** to **MI-10** were not able to inhibit the growth of most of the tested fungi. Actually, only the growth of *A. nomius* and *A. parasiticus* was affected by high concentrations of **MI-8** and **MI-10**. Additionally, the assayed mesoionic compounds showed low antimicrobial activity against *P. citrinum*. Table 1 summarizes the results. Itraconazole, an antifungal tested as a reference compound, showed MIC of 0.12 μg/ml for all *Aspergillus* species and *P. citrinum*, and, for *F. verticillioides,* the MIC value was 4 μg/ml. The minimum fungicidal concentrations (MFC) of the mesoionic compounds were only determined for *F. verticillioides*, which was the most sensitive fungus. Additionally, the most active substances, **MI-1**, **MI-2**, **MI-3**, **MI-5**, **MI-11**, and **MI 14**–**16**, were assayed to assess the MFC values. Our results indicated a fungicidal effect for all compounds tested (Table 1), since no visual fungal growth was observed in the MFC, that correspond to a killing activity of approximately 99% as established by Espinel-Ingroff and co-workers [20].

**Figure 3:**
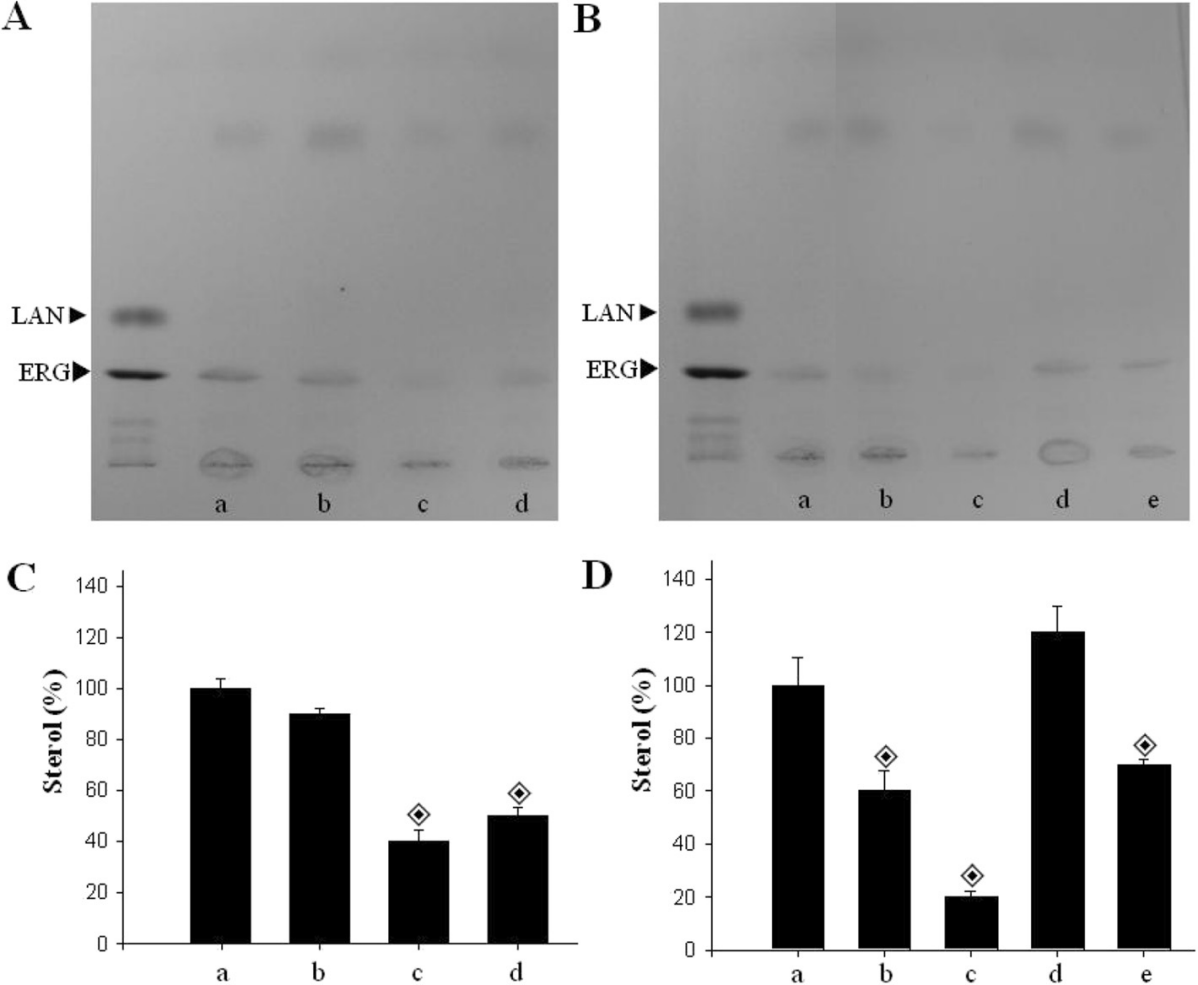
**The effect of mesoionic compounds on ergosterol production by** ***F. verticillioides*** **. (A)** Conidia were incubated at 26°C for 48 h in RPMI 1640 medium in the absence (a, control system) or presence of 3.9 μg/mL (c) or 7.8 μg/mL (d) of the mesoionic compounds **MI-16**. **(B)** Fungal cells not treated (a, control) or treated with 7.8 μg/mL (b) or 15.6 μg/mL (c) of **MI-2** or with 3.9 μg/mL (d) or 7.8 μg/mL (e) of **MI-11**. After treatment, total lipids were extracted, and the obtained neutral lipids were applied to HPTLC plates. Ergosterol (ERG) and lanosterol (LAN) were used as sterol standards, as indicated by arrows. The use of 1% DMSO as eluent for the compounds did not alter the ergosterol levels (b, panel **A)**. Densitometric quantifications **(C and** **D)** of the bands corresponding to panels **A** and **B**, respectively. Graphical representation of HPTLC data, which were analyzed using the Image J software. The sterol content of the control was set as 100%. Symbols denote significant differences (◈, P <0.05 Student’s t test) when compared to control cells (no treatment).

Of the mesoionic compounds that presented the best antifungal activity, and fungicidal effect against *F. verticillioides*, the abilities of **MI-2**, **MI-11** and **MI-16** to change the amount of ergosterol in the total lipid content at the MIC and sub-MIC concentrations were evaluated using High Performance Thin Layer Chromatography (HPTLC). The treatments using 7.8 and 15.6 μg/ml **MI-2** (Figure 3B and D) caused decreases in the ergosterol content of 40% and 80%, respectively. In addition, **MI-11** (Figure 3B and D) 7.8 μg/ml inhibited 30% the level of sterol, while 3.9 μg/ml of this compound was not able to affect fungal lipid. Whereas, *F. verticillioides* ergosterol was reduced 60% and 50% after treatment with **MI-16** compound (Figure 3A and C) at 3.9 and 7.8 μg/ml, respectively. Although the mechanism of action of these substances is not well established, our results indicate that reduction in the sterol content could be involved in the growth inhibition of *F. verticillioides*. Jin and co-authors reported a decreased ergosterol content in *Gibberella zeae* (anamorph *Fusarium graminearum*) when exposed to methyl-sulphone derivatives containing the thiadiazole moiety during an investigation of the mechanism of the antifungal effects of those compounds. The best result revealed a 55.34% inhibition of ergosterol biosynthesis [21].

Our results suggest that these mesoionic compounds can act similar to the class of azole antifungal agents in inhibiting ergosterol biosynthesis [22]. The chemical structures of mesoionic compounds are similar to azole systems, having a pentacyclic ring with two or three nitrogen atoms. In the mesoionic ring, an additional two nitrogen atoms and one sulfur atom is present.

The deficiency in sterol content affects important properties of the cell membrane, leading to increased fluidity and membrane permeability that results in loss of the selective passage of substances from the cell, an intense disruption of the fungal cells and possibly death [23]. However, the data do not exclude the possibility that these compounds could interfere with other metabolic pathways that are important for fungal proliferation. Additional experiments must be conducted to confirm these results and clarify the mechanisms of action of these synthetic antifungal substances.

This study was focused on the screening of mesoionic compounds for antifungal activity (*Aspergillus* spp*.*, *F. verticillioides* and *P. citrinum*). Experiments are in progress to determine whether other species of genus *Fusarium* are also sensitive to mesoionic compounds.

## Conclusions

The high contamination of important agricultural commodities with mycotoxigenic fungi and the microbial resistance toward available drugs encourages us to study the antifungal activity of mesoionic compounds. Our results showed that mesoionic compounds, mainly the methoxy-substituted derivatives, were more active in impairing the growth and sterol content of *F. verticillioides*. Although the mechanism of action of these compounds is not well understood, our results indicated that a reduction in the sterol content could be involved in the growth inhibition of *F. verticillioides*. In this context, our data corroborate the supposition that this class of heterocyclic compounds has the potential to be used to minimize and/or control mycotoxin-producing fungi.

## Methods

### Preparation of the synthetic substances

The mesoionic compounds (**MI**) were synthesized as previously reported [9]. Briefly, the substituted benzaldehydes (0.35 mmol) and thiosemicarbazides (0.35 mmol) were mixed in the presence of thionyl chloride and a few drops of 1,4-dioxane. The mixture was then submitted to microwave irradiation for 5 min in an open vessel. After this time, the mixture was added to 1,4-dioxane and left to stand overnight at room temperature. The obtained products were filtered, washed with ice-cold water and purified by recrystallization from chloroform:ethanol (60:40, v/v). All products were characterized by routine spectroscopic techniques, such as infrared, mass, ^1^H and ^13^C NMR. Table 2 summarize the main spectroscopic parameters for synthesized compounds.

**Table 2:** **Main characteristic values of infrared (IR) and** ^**1**^ **H and** ^**13**^ **C Nuclear Magnetic Resonance (NMR) of synthesized mesoionic compounds**

**Compound**	**IR**	^**1**^ **H NMR**	^**13**^ **C NMR**
	**ν (cm** ^**−1**^ **)**	**(δ)**	**(δ)**
**MI-1**	3432 (N-H); 3056 (C-H); 2670 (C = NH^+^); 1567 (C = N); 1330 (C-S)	7.95 (d, H-α), 7.83-7.19 (m, 15 H), 7.06 (d, H-β)	159.2 (C-5), 147.8 (C-2), 137.0 (C-α), 133.9-118.7 (18 C), 111.6 (C-β)
**MI-2**	3432 (N-H); 3025 (C-H); 2969 (C-H); 2696 (C = NH^+^); 1567 (C = N); 1311 (C-S)	7.89 (d, H-α), 7.76-7.01 (m, 14 H), 6.97 (d, H-β), 3.84 (s, 3H, OCH_3_)	163.3 (C-4’), 162.5 (C-5), 158.4 (C-2), 148.3 ( C-α), 138.7-114.8 (17 C), 108.8 (C-β), 55.6 (OCH_3_)
**MI-3**	3432 (N-H); 3048 (C-H); 2721 (C = NH^+^); 1571 (C = N); 1519 (C-NO_2_); 1342 (C-S)	8.04 (d, H-α), 7.80-7.39 (m, 14 H), 7.33 (d, H-β)	162.0 (C-5), 159.7 (C-2), 148.6 (C-4’), 144.7 ( C-α), 140.0-118.7 (17 C), 115.5 (C-β)
**MI-4**	3423 (N-H); 3054 (C-H); 2925 (C-H); 2786 (C = NH^+^); 1565 (C = N); 1295 (C-S)	7.92 (d, H-α), 7.77-7.10 (m, 14 H), 7.05 (d, H-β), 3.81 (s, 3H, OCH_3_)	162.3 (C-4’), 159.2 (C-5), 158.6 (C-2), 144.6 (C-α), 138.3-118.9 (17 C), 111.40 (C-β), 55.5 (OCH_3_)
**MI-5**	3425 (N-H); 3062 (C-H); 2923 (C-H); 2852 (C = NH^+^); 1565 (C = N); 1378 (C-S)	10.24 (s, 1H, N-H), 7.90 (d, H-α), 7.73-7.13 (m, 14 H), 6.74 (d, H-β), 3.05–2.94 (m, 6H, NCH_3_)	164.9 (C-4’), 155.1 (C-5), 151.8 (C-2), 140.0 (C-α), 138.7-113.1 (17 C), 104.0 (C-β), 40.2 (N-CH_3_)
**MI-6**	3435 (N-H); 2997 (C-H); 2582 (C = NH^+^); 1541 (C = N); 1338 (NO_2_); 1627, 1585, 1338 (C-S)	12.58 (s, 1H, N-H), 8.25-7.22 (m, 14 H), 8.10 (d, 1H, H-α), 7.25 (d, 1H, H-β)	159.8 (C-5), 148.5 (C-2), 144.5 (C-α), 139.9-118.8 (17 C), 115.5 (C-β)
**MI-7**	3431 (N-H); 3043 (C-H); 2929 (C-H); 2717 (C = NH^+^); 1512 (C = N); 1342 (C-S); 1109 (O-CH_3_)	12.73 (s, 1H, N-H), 8.24-6.99 (m, 14 H), 8.09 (d, 1H, H-α), 7.24 (d, 1H, H-β), 3.73 (s, 3H, OCH_3_)	161.3 (C-5), 156.0 (C-2), 148.5-114.6 (17 C), 144.2 (C-α), 115.5 (C-β), 55.3 (OCH_3_)
**MI-8**	3431 (N-H); 3039 (C-H); 2922 (C-H); 2746 (C = NH^+^); 1523 (C = N); 1340 (C-S)	12.58 (s, 1H, N-H), 8.25-7.22 (m, 14 H), 8.10 (d, 1H, H-α), 7.25 (d, 1H, H- β), 2.27 (s, 3H, CH_3_)	159.8 (C-5), 148.5 (C-2), 144.5 (C-α), 139.9-118.8 (17 C), 115.5 (C-β), 20.5 (CH_3_)
**MI-9**	3432 (N-H); 3037 (C-H); 2663 (C = NH^+^); 1540 (C = N); 1309 (C-S); 948, 669 (C-Br)	12.58 (s, 1H, N-H), 8.25-7.22 (m, 14 H), 8.10 (d, 1H, Hα), 7.25 (d, 1H, Hβ)	159.8 (C-5), 148.5 (C-2), 144.5 (C-α), 139.9-118.8 (17 C), 115.5 (C-β)
**MI-10**	3419 (N-H); 3047 (C-H); 2723 (C = NH^+^); 1569 (C = N); 1319 (C-S)	7.72-7.08 (m, 15 H)	163.8 (C-5), 160.5 (C-2), 138,6-118.3 (18 C).
**MI-11**	3425 (N-H); 3048 (C-H); 2935 (C-H); 2665 (C = NH^+^); 1567 (C = N); 1309 (C-S)	12.90 (s, 1H, N-H), 7.70-7.25 (m, 14 H), 3.86 (s, 3H, OCH_3_)	163.0 (C-5), 159.5 (C-2), 138.3- 114.7 (17 C), 55,5 (OCH_3_)
**MI-12**	3434 (N-H); 3043 (C-H); 2721 (C = NH^+^); 1573 (C = N); 1537 (C-NO_2_); 1348 (C-S)	7.71-7.00 (m, 14 H)	160.8 (C-5), 149.2 (C-2), 138.0 (C-4’), 137.0-118.2 (17 C)
**MI-13**	3438 (N-H); 3050 (C-H); 2902 (C-H); 2642 (C = NH^+^); 1567 (C = N); 1311 (C-S)	9.76 (s, 1H, N-H), 7.62-6.60 (m, 13 H), 6.08 (s, 2H, OCH_2_O)	165.1 (C-5), 161.5 (C-2), 152.7-110.4 (17 C), 149.0 (C-4’), 103.9 (OCH_2_O)
**MI-14**	3435 (N-H); 3055 (C-H); 2719 (C = NH^+^); 1579 (C = N); 1543 (C-NO_2_); 1340 (C-S)	13.62 (s, 1H, N-H), 8.32-7.52 (m, 14 H)	165.8 (C-5), 160.4 (C-2), 144.0 (C-4”’), 142.5-118.7 (17 C)
**MI-15**	3435 (N-H); 3184 (C-H); 3039 (C-H); 2736 (C = NH^+^); 1508 (C = N); 1249 (C-S)	12.30 (s, 1H, N-H), 7.66-7.00 (m, 14 H), 3.73 (s, 3H, OCH_3_)	161.3 (C-5), 156.1 (C-2), 148.5 (C-4”’), 144.2-114.6 (17 C), 55.3 (OCH_3_)
**MI-16**	3433 (N-H); 3041 (C-H); 2914 (C-H); 2767 (C = NH^+^); 1562 (C = N); 1220 (C-S)	12.73 (s, 1H, N-H), 7.67-7.21 (m, 14 H), 2.49 (s, 3H, CH_3_)	163.9 (C-5), 160.4 (C-2), 137.7-118;4 (17 C), 133.2 (C-4”’), 20.5 (CH_3_).

All commercial reagents for the synthesis of the mesoionic compounds were obtained from Sigma-Aldrich or Acros Co. and used without any further purification.

For the assays, these synthetic substances were solubilized in DMSO (Merck):Tween20® (Merck):RPMI 1640 (Invitrogen, USA) diluted at a 1:1:8 ratio. The synthetic culture medium, RPMI 1640, with L-glutamine but without sodium bicarbonate, was buffered with 3-(*N*-morpholino) propanesulfonic acid (MOPS (Merck), final concentration 0.165 mol l^−1^, pH 7.0).

### Fungal isolates

The fungal strains were obtained from the Mycological *Trichocomaceae* Collection of the Oswaldo Cruz Institute, Fiocruz, Rio de Janeiro. *Aspergillus flavus* MCT 00040, *A. nomius* MCT 00328, *A. ochraceus* MCT 00435, *A. parasiticus* MCT 00334, *Fusarium verticillioides* MCT 00177 and *Penicillium citrinum* MCT 00151 were rehydrated and activated in Sabouraud dextrose agar (SDA) medium and incubated for 7 days at 25°C.

### Culture conditions

For conidia formation, the cultures were grown in potato dextrose agar (PDA) medium for 7 days at 35°C, with the exception of *A. ochraceus,* which was incubated for 7 days at 25°C, and *F. verticillioides,* which was maintained at 35°C for 48 h and then at 25°C until day 7 of incubation. *P. citrinum* was cultured on Czapek-Dox agar, pH 5.5, at 25°C for 7 days. Conidia were collected from the culture plates by scraping the cells with a Pasteur pipette. The conidia suspensions were recovered with saline supplemented with 1% Tween-20. Then, the cell suspension was transferred to a centrifuge tube, allowed to rest for 10 min and then centrifuged at 4000 g for 10 min. The pellet, consisting of conidia, was washed twice with saline, and the final cell concentration was adjusted spectrophotometrically [13].

### Determination of the minimal inhibitory concentration (MIC)

Antifungal susceptibility testing was performed as described in the M38-A document of the Clinical and Laboratory Standards Institute [13] for filamentous fungus. Briefly, the broth microdilution test was performed in a 96-well microtiter plate containing RPMI 1640 medium (Invitrogen, USA) at pH 7.0 and buffered with 0.165 mol l^−1^ of 3-(*N*-morpholino) propanesulfonic acid (MOPS) (Merck, Darmstad, Germany). The synthetic substances were diluted to obtain final concentrations ranging from 3.9 to 500 μg/ml, and the maximum concentration of organic solvent was 2.5%. *Aspergillus* spp. and *F. verticillioides* conidia were inoculated into the appropriate wells at a final concentration of 0.4 - 5 × 10^4^ CFU ml^−1^. A control system was performed in a similar way without the addition of compounds. The minimum inhibitory concentration (MIC) of each drug was determined visually after incubation at 35°C for 48 h. The MIC was considered the lowest concentration of the substance able to completely inhibit (100%) visible growth of the fungus. Itraconazole (Sigma-Aldrich Chemical Co., Missouri, USA) was used as a reference compound at final concentrations ranging from 16 to 0.12 μg/ml. Each experiment was performed in triplicate.

### Determination of the minimal fungicidal concentration (MFC)

The *in vitro* fungicidal activities of the compounds for *F. verticillioides* were determined as described by Espinel-Ingroff and co-workers [20]. Briefly, prior to spectrophotometric reading of the microtiter plates that were used in the broth microdilution tests, the contents of each well that showed complete growth inhibition in the MIC test were homogenized, and an aliquot from each well was transferred onto drug-free SDA plates. The plates were incubated at 30°C for 9 days, and the MFC was defined as the lowest drug dilutions that yielded fewer than three colonies (approximately 99 to 99.5% killing).

### Effect of the mesoionic compounds in decreasing the amount of ergosterol in the total lipid content

The abilities of sub-inhibitory and inhibitory concentrations of **MI-16** (3.9 and 7.8 μg/ml), **MI-2** (7.8 and 15.6 μg/ml) and **MI**-**11** (3.9 and 7.8 μg/ml) to decrease the amount of ergosterol in the total lipid content of *F. verticillioides* were evaluated. Conidia were grown in PDA, and a cell suspension was obtained as detailed in the culture conditions section. Conidia were washed twice with saline and once with RPMI, and the final cell concentration was adjusted to 1 × 10^7^ cells/ml. Then, conidia were incubated at 26°C in RPMI medium in the presence or absence (control) of mesoionic compounds. After 48 h, conidia were washed 3 times with 0.85% sodium chloride and submitted to total lipid extraction using chloroform:methanol (2:1, 1:1, and 1:2) mixtures [24]. Combined extracts were dried, and Folch partition was performed [25]. The neutral lipids (lower phase) were dried under streaming N_2_, redissolved in chloroform and spotted onto high performance thin-layer chromatography (HPTLC) plates. Chromatography was performed on silica gel 60 plates (Sigma Chemical Co., Missouri, USA), and the plates were developed with hexane:diethyl ether:acetic acid (60:30:1.5). The spots were visualized after dipping the plate in chemical reagent (50 mg of iron chloride, 5 ml of acetic acid, 5 ml of sulfuric acid and 90 ml of distilled water) and subsequent heating [26]. Ergosterol (4 μg) and lanosterol (1 μg), which were purchased from Sigma Chemical Co. (Missouri, USA), were used as sterol standards. Densitometric quantification of ergosterol was performed using the free Image J software [27]. This experiment was performed three times, with similar results obtained from three independent cultures/extractions. The concentrations of mesoionic compounds tested in this experiment did not affect the viability of *F. verticillioides* conidia.

### Statistical analysis

All experiments were performed in triplicate, in three independent experimental sets. The data were analyzed statistically by means of Student’s t-test using EPI-INFO 6.04 (Database and Statistics Program for Public Health) computer software.
